# Bispectral index monitoring reduces sedative and vasopressor requirements during percutaneous tracheostomy

**DOI:** 10.1186/cc9768

**Published:** 2011-03-11

**Authors:** P Hampshire, A Guha, I Welters, J Murphy, L Poole

**Affiliations:** 1 Royal Liverpool and Broadgreen University Hospital, Liverpool, UK

## Introduction

Bispectral index (BIS) monitoring measures depth of anaesthesia, using electroencephalography (EEG). It has been validated against sedation scales used in intensive care. We hypothesized that using BIS during percutaneous tracheostomies would reduce sedation doses, resulting in fewer episodes of haemodynamic instability.

## Methods

Patients undergoing percutaneous tracheostomy were randomised to the control or intervention groups. Norepinephrine was administered to prevent a fall of more than 20% in mean arterial blood pressure. Patients in the control group were sedated with a propofol infusion at a dose chosen by the operator. All personnel performing the tracheostomy were blinded to the BIS score. In the intervention group, patients were sedated with a propofol infusion adjusted so that the BIS was maintained between 45 and 60. Patients with encephalopathy or brain injury, and patients who had received sedative drugs other than alfentanil and propofol were excluded. All patients or their advocates gave written, informed consent. The primary outcome was the number of episodes of haemodynamic instability. Secondary outcomes were the dose of propofol administered to patients, BIS scores, time of recovery from sedation, total norepinephrine administered to patients, and time taken to do the procedure.

## Results

Twenty patients entered the study. Results are presented as mean ± SD. There was no significant difference in the incidence of hypotension (4.5 ± 6.8 events and 5.6 ± 6.9 events in control and intervention groups, respectively, *P *= 0.25). There were fewer episodes of hypertension in the intervention group (2.5 ± 4.6 events in the control group and 0.9 ± 2.2 events in the intervention group) (*P *= 0.12). The dose of propofol and norepinephrine dose were lower in the intervention group: 5.4 mg/kg/hour cf. to 6.8 mg/kg/hour for propofol (*P *= 0.21); 0.05 μg/kg/hour cf. to 0.09 μg/kg/hour for norepinephrine (*P *= 0.14). The mean time to waking was significantly shorter in the intervention group (54 minutes) as compared with that in the control group (96 minutes), *P *= 0.04.

## Conclusions

BIS monitoring did not significantly reduce sedation requirements, or improve haemodynamics during percutaneous tracheostomy, although there was a trend to both reduced sedation requirements and improved haemodynamic stability. The time to waking was significantly reduced.

